# Novel biomarkers of mitochondrial dysfunction in Long COVID patients

**DOI:** 10.1007/s11357-024-01398-4

**Published:** 2024-11-04

**Authors:** Titanilla Szögi, Barbara N. Borsos, Dejana Masic, Bence Radics, Zsolt Bella, Andrea Bánfi, Nóra Ördög, Csenge Zsiros, Ágnes Kiricsi, Gabriella Pankotai-Bodó, Ágnes Kovács, Dóra Paróczai, Andrea Lugosi Botkáné, Béla Kajtár, Farkas Sükösd, Andrea Lehoczki, Tamás Polgár, Annamária Letoha, Tibor Pankotai, László Tiszlavicz

**Affiliations:** 1 Department of Pathology, Albert Szent-Györgyi Medical School, University of Szeged, Szeged, Hungary; 2 Competence Centre of the Life Sciences Cluster of the Centre of Excellence for Interdisciplinary Research, Development and Innovation, University of Szeged, Szeged, Hungary; 3 Department of Oto-Rhino- Laryngology and Head-Neck Surgery, Albert Szent-Györgyi Medical School, University of Szeged, Szeged, Hungary; 4 Department of Pediatrics and Pediatric Health Center, Albert Szent-Györgyi Medical School, University of Szeged, Szeged, Hungary; 5 Department of Internal Medicine, Albert Szent-Györgyi Medical School, University of Szeged, Szeged, Hungary; 6 Pulmonology Clinic, Albert Szent-Györgyi Medical and Pharmaceutical Centre, University of Szeged, Szeged, Hungary; 7 Department of Pathology, University of Pécs Medical School, Pécs, Hungary; 8 Doctoral College, Health Sciences Program, Semmelweis University, Budapest, Hungary; 9 Institute of Preventive Medicine and Public Health, Semmelweis University, Budapest, Hungary; 10 Genome Integrity and DNA Repair Core Group, Hungarian Centre of Excellence for Molecular Medicine (HCEMM), Hungarian Centre of Excellence for Molecular Medicine, Szeged, Hungary; 11 Institute of Biophysics, HUN-REN Biological Research Centre, Szeged, Hungary; 12 Theoretical Medicine Doctoral School, University of Szeged, Szeged, Hungary

**Keywords:** Mitochondria, Post-COVID, Mitophagy, Oxidative damage, mtDNA

## Abstract

Coronavirus disease 2019 (COVID-19) can lead to severe acute respiratory syndrome, and while most individuals recover within weeks, approximately 30–40% experience persistent symptoms collectively known as Long COVID, post-COVID-19 syndrome, or post-acute sequelae of severe acute respiratory syndrome coronavirus 2 (SARS-CoV-2) infection (PASC). These enduring symptoms, including fatigue, respiratory difficulties, body pain, short-term memory loss, concentration issues, and sleep disturbances, can persist for months. According to recent studies, SARS-CoV-2 infection causes prolonged disruptions in mitochondrial function, significantly altering cellular energy metabolism. Our research employed transmission electron microscopy to reveal distinct mitochondrial structural abnormalities in Long COVID patients, notably including significant swelling, disrupted cristae, and an overall irregular morphology, which collectively indicates severe mitochondrial distress. We noted increased levels of superoxide dismutase 1 which signals oxidative stress and elevated autophagy-related 4B cysteine peptidase levels, indicating disruptions in mitophagy. Importantly, our analysis also identified reduced levels of circulating cell-free mitochondrial DNA (ccf-mtDNA) in these patients, serving as a novel biomarker for the condition. These findings underscore the crucial role of persistent mitochondrial dysfunction in the pathogenesis of Long COVID. Further exploration of the cellular and molecular mechanisms underlying post-viral mitochondrial dysfunction is critical, particularly to understand the roles of autoimmune reactions and the reactivation of latent viruses in perpetuating these conditions. This comprehensive understanding could pave the way for targeted therapeutic interventions designed to alleviate the chronic impacts of Long COVID. By utilizing circulating ccf-mtDNA and other novel mitochondrial biomarkers, we can enhance our diagnostic capabilities and improve the management of this complex syndrome.

## Introduction

The emergence of coronavirus disease 2019 (COVID-19), caused by the severe acute respiratory syndrome coronavirus 2 (SARS-CoV-2), has precipitated a global health crisis with enduring implications. As of the latest updates, COVID-19 has affected over 775 million individuals worldwide, resulting in more than 7 million deaths across various countries and territories [1]. The mortality rate for COVID-19 differs significantly by age, with older adults, especially those with underlying health conditions, experiencing disproportionately higher rates of fatalities [2–5]. The pandemic has seen multiple waves, driven by the emergence of virus variants, each varying in transmissibility and virulence [6, 7]. Despite extensive vaccination efforts, which have seen billions of vaccine doses administered globally, the virus continues to impact populations, healthcare systems, and economies.

While the majority of affected individuals recover from the acute respiratory syndrome within a few weeks, approximately 30–70% of those infected experience persistent and debilitating symptoms collectively termed Long COVID, post-COVID-19 syndrome, or post-acute sequelae of SARS-CoV-2 infection (PASC) [3, 8–26]. Chronic fatigue is consistently identified as the most common and debilitating symptom reported by survivors, as demonstrated by various cross-sectional and cohort studies [18, 27–31]. Individuals affected by Long COVID often experience a broad range of additional symptoms, including dyspnea, joint pain, sleep problems, mood disorders such as depression and anxiety [32], headaches, dizziness, cognitive issues commonly referred to as “brain fog,” and cardiac symptoms [18]. These symptoms can persist for months and significantly impair quality of life. The National Institute for Health and Care Excellence categorizes PASC as ongoing symptomatic COVID-19 for individuals whose symptoms persist between 4 and 12 weeks following the initial onset of acute symptoms or as post-COVID-19 syndrome for those whose symptoms continue beyond 12 weeks [18, 33]. In contrast, the World Health Organization describes PASC as a condition affecting individuals with a suspected or confirmed SARS-CoV-2 infection who experience lasting symptoms for a minimum of 2 months and where these symptoms cannot be attributed to another underlying medical condition [9, 34].

Long COVID presents a complex clinical picture that implicates multiple organ systems. Emerging evidence suggests mitochondrial dysfunction as a central component of this syndrome [35–49]. Mitochondria, essential for energy production and cellular metabolism, are particularly vulnerable to SARS-CoV-2 infection [36]. The virus may hijack and reprogram mitochondrial function or inflict direct damage through various mechanisms during and potentially after infection [36]. Such disruptions lead to altered energy metabolism, which is believed to contribute to the fatigue, cognitive impairments, and muscular weaknesses commonly observed in Long COVID patients [35, 36].

The primary goal of this study was to investigate novel biomarkers of mitochondrial dysfunction in Long COVID patients and their correlation with persistent symptoms, particularly chronic fatigue. To achieve this, we conducted a series of comparative analyses between post-COVID-19 patients and controls. Utilizing transmission electron microscopy, we inspected nasal mucosal and bronchial biopsy samples to identify and characterize mitochondrial structural abnormalities and their association with Long COVID symptoms. We quantified the levels of proteins crucial to mitochondrial dynamics—specifically autophagy-related 4B cysteine peptidase (ATG4B), mitofusin 2 (MFN2), and dynamin-related protein 1 (DRP1). Elevated levels of these proteins might indicate ongoing mitochondrial dysfunction or compensatory responses within affected cells. Additionally, measuring superoxide dismutase 1 (SOD1) protein levels provided insights into the oxidative stress status of these patients. By assessing the circulating cell-free mitochondrial DNA (ccf-mtDNA) in blood plasma, we evaluated the integrity and functionality of mitochondrial recycling processes in post-COVID-19 patients. Through these objectives, the study sought to validate the hypothesis that persistent mitochondrial dysfunction significantly contributes to the chronic symptoms of Long COVID.

## Materials and methods

### Cohort characteristics

For the measurement of circulating cell-free mitochondrial DNA (ccf-mtDNA), the study enrolled 32 post-COVID-19 (PC) patients and 31 healthy volunteers, with median ages of 46 and 44 years, respectively. The most prevalent symptoms among PC patients included disorders of smell and taste—specifically anosmia, hyposmia, dysosmia, ageusia, hypogeusia, and dysgeusia. Additionally, these patients frequently reported impaired memory, fatigue, paresthesia, cardiac arrhythmias, tachycardia, dyspnea, thoracic and joint disorders, urticaria, and other dermatological issues (Table 1, left part). The selection of the PC patients was carried out as described by Pavli et al. [50].

**Table 1:** Cohort characteristics for transmission electron microscopy (TEM) and circulating cell-free mitochondrial DNA (ccf-mtDNA) studies

	Cohort characteristics				
		ccf-mtDNA		TEM	
		PC	C	PC	C
Age	Median age (years)	46	44	28	10
Sex distribution	Female (number of participants)	24	21	3	1
	Male (number of participants)	8	10	2	4
Symptoms	Anosmia/Hyposmia/Dysosmia	16	–	5	–
	Ageusia/Hypogeusia/Dysgeusia	8	–	1	–
	Impaired memory	2	–	–	–
	Fatigue	2	–	1	–
	Paresthesia	2	–	–	–
	Cardiac arrhythmia	1	–	–	–
	Tachycardia	1	–	–	–
	Dyspnea	1	–	–	–
	Thoracic disorders	1	–	–	–
	Joint disorders	1	–	–	–
	Urticaria	1	–	–	–
	Other respiratory disorder	–	–	4	–
	Other dermatological condition	1	–	–	–

For transmission electron microscopy (TEM) analysis, nasal mucosal and bronchial biopsy samples were collected from five PC patients (median age 28 years) and five controls who exhibited no post-COVID-19 symptoms but were diagnosed with secondary ciliary dyskinesia (median age 10 years). The primary symptoms of PC patients were smell disorders—anosmia, hyposmia, and dysosmia. Other reported symptoms included taste disorders—ageusia, hypogeusia, and dysgeusia—fatigue, and various respiratory conditions (Table 1, right part).

### Sample preparation and post-embedding for immunohistochemistry

All cases of human nasal mucosa and bronchial biopsy were previously diagnosed and collected from the archives of the University of Szeged. All specimens were initially preserved in a 3% glutaraldehyde solution supplemented with dextran. Upon arrival at the Department of Pathology, both control (*n* = 5) and PC (*n* = 5) samples underwent a post-fixation in a fresh 3% glutaraldehyde solution. The samples were then rinsed in phosphate-buffered saline (PBS) and fixed for 1 h in 2% osmium tetroxide. The specimens were dehydrated through a graded series of ethanol concentrations, followed by rinsing in uranyl acetate and acetone. Subsequently, they were embedded in Embed812 resin (Electron Microscopy Sciences; Hatfield, PA, USA). Ultrathin Sections (70 nm) were prepared using an Ultracut S ultra-microtome (Leica, Wetzlar, Germany) and mounted on copper grids [51].

Post-embedding sections were blocked with 1% bovine serum albumin for 20 min and then washed three times in PBS. They were incubated with primary antibodies at room temperature for either 1 h or 3 h, depending on the specific antibody (Table 2). After washing in PBS, sections were incubated with appropriate secondary antibodies—anti-rabbit (for DRP1, MFN2, ATG4B, FIS1, and LDH) or anti-mouse (for MFN1)—for 3 h at room temperature (Table 3). Finally, sections were counterstained with 0.25% uranyl acetate (Electron Microscopy Sciences, Hatfield, PA, USA) and 3% lead citrate (Leica, Wetzlar, Germany) to enhance contrast [52].

**Table 2:** Primary antibodies used in immunohistochemistry for TEM

Antibody	Target protein	Host species	Dilution; incubation time	Catalog number	Supplier
Anti-DRP1	Dynamin-related protein 1	Rabbit	1:25; 1 h	ab184247	Abcam, Cambridge, UK
Anti-MFN1	Mitofusin 1	Mouse	1:50; 1 h	MA5-36,240	Invitrogen, Waltham, Massachusetts, USA
Anti-MFN2	Mitofusin 2	Rabbit	1:25; 3 h	ab219730	Abcam, Cambridge, UK
Anti-ATG4B	Autophagy-related protein 4B	Rabbit	1:50; 1 h	710,915	Invitrogen, Waltham, Massachusetts, USA
Anti-FIS1	Mitochondrial fission 1 protein	Rabbit	1:800; 1 h	ab229969	Abcam, Cambridge, UK
Anti-SOD1	Superoxide dismutase 1	Mouse	1:25; 1 h	MA1-105	Invitrogen, Waltham, Massachusetts, USA
Anti-LDH	Lactate dehydrogenase	Rabbit	1:25; 1 h	ab52488	Abcam, Cambridge, UK

**Table 3:** Secondary antibodies used in immunohistochemistry for TEM. Dilutions are provided by the supplier and optimized for use in TEM to ensure specific binding and minimal background. Proper handling and storage of antibodies were ensured as per supplier recommendations to maintain activity

Secondary antibodies	Host species	Size of colloidal gold particles	Dilution	Catalog number	Supplier
Anti-mouse IgG	goat	10 nm	1:20	G3779	Sigma-Aldrich, St. Louis, MO, USA
Anti-rabbit IgG	goat	18 nm	1:40	111–215-144	Sigma-Aldrich, St. Louis, MO, USA

### Quantification of immunohistochemistry

For each sample, five cells were imaged using a JEOL JEM 1400 TEM (JEOL; Tokyo, Japan) at magnifications of × 12,000 and × 20,000. Images were captured using TEM Center software (JEOL; Tokyo, Japan). To quantify the data, each image was analyzed using the point counting grid method with Image-Pro Plus software (Media Cybernetics, Rockville, Maryland, USA). A 20 × 20 grid was superimposed over each image, and intersections of grid points with mitochondria were counted. Additionally, the number of gold particles intersected by the grids within mitochondrial regions was tallied. This mitochondrial-associated gold particle count was then normalized to the delimited mitochondrial area for each image.

Due to the non-normal distribution of the data, statistical analysis was performed using the nonparametric Mann–Whitney *U* test. All statistical evaluations were executed using SPSS software (IBM SPSS Statistics 29; New York, USA). To visually represent the data distribution, violin plots were generated using the Flourish online tool [53].

### Plasma isolation

Blood samples were collected from PC patients and healthy individuals using 10-ml cell-free DNA BCT tubes (Streck). The tubes were gently inverted ten times to mix and then centrifuged for 10 min at 2000 rpm at 4 °C. The upper plasma layer was carefully transferred to a sterile tube and centrifuged again for 10 min at 4500 rpm at 4 °C to eliminate any residual cellular components. Two milliliters of the clarified plasma was then used for each subsequent isolation procedure.

### Ccf-DNA isolation and mtDNA content measurement

The QIAamp MinElute ccf-DNA Mini Kit (Qiagen) was employed for the isolation of circulating cell-free DNA (ccf-DNA) following the manufacturer’s protocol. The concentration of isolated ccf-DNA was determined using a Qubit 4 fluorometer (Invitrogen). For each quantitative PCR (qPCR) reaction, 0.5 ng of ccf-DNA was used. Relative quantification of mitochondrial DNA (mtDNA) content was performed using qPCR (Rotor-Gene Q, Qiagen) with specific primers, employing cyclophilin B as an internal control to ensure accurate and consistent results.

### Statistical analysis of ccf-mtDNA content measurements

To visualize the discriminating potential of the measured ccf-mtDNA, a heat map was generated using the ClustVis online tool [54]. Statistical differences in ccf-mtDNA content between PC patients and healthy volunteers were assessed using independent samples *t*-tests performed with SPSS software (IBM SPSS Statistics 29; New York, USA). Additionally, violin plots were created using the Flourish online tool to provide a detailed view of the data distribution [53].

To evaluate the diagnostic potential of the ccf-mtDNA measurements, receiver operating characteristic (ROC) curves and the corresponding area under the curve (AUC) values were calculated using SPSS software. These analyses help determine the effectiveness of ccf-mtDNA levels in distinguishing between PC patients and healthy controls.

### Ethics statement

This study received ethical approval from the Institutional Review Board of the Albert Szent-Györgyi Clinical Centre at the University of Szeged (approval number 100/2022-SZTE RKEB). All procedures performed in studies involving human participants were in accordance with the ethical standards of the institutional and national research committee and with the 1964 Helsinki Declaration and its later amendments.

## Results

### Structural and functional mitochondrial impairment in post-COVID-19 syndrome

**Fig. 1:**
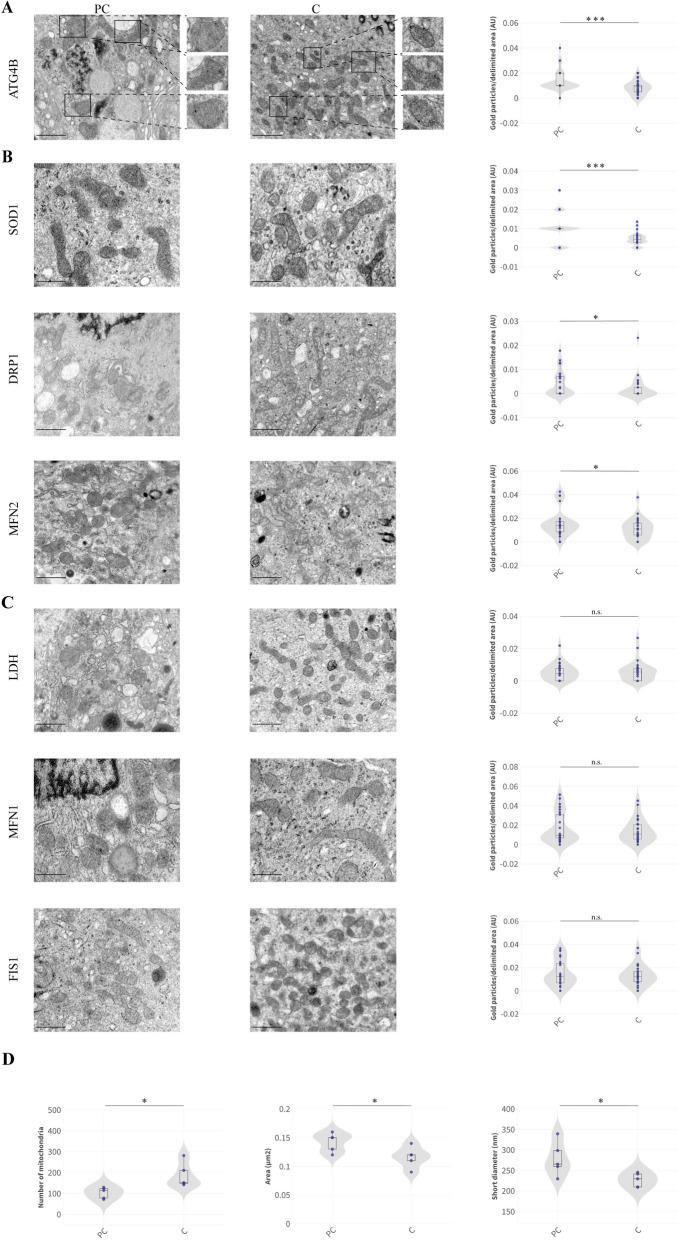
Analysis of mitochondrial morphology and expression of specific proteins related to mitochondrial function in patients with post-COVID-19 (PC) syndrome and control participants by TEM. Mitochondrial morphology and immunodetection of proteins associated with mitochondrial function in patients (first column) and control (C) participants (second column). Protein markers analyzed include **A** ATG4B; **B** SOD1, DRP1, and MFN2; and **C** LDH, MFN1, and FIS1. In the third column, violin plots quantitatively present the immunodetection results corresponding to the protein markers listed in the same row. Statistical significance between PC and C samples is denoted by asterisks: *p < 0.05, ***p < 0.001. “ns” indicates no significant differences (*p* > 0.05). **D** Quantitatively presents the analysis of mitochondrial morphology and copy number differences in PC patients

Using TEM, we examined mitochondrial ultrastructure in nasal mucosal and bronchial needle biopsies from five PC and five control patients. TEM analysis revealed distorted mitochondrial integrity in PC patients, characterized by dilated and washed-out cristae and enlarged mitochondria compared to controls. Additionally, protein levels related to mitochondrial dynamics were quantified. Mitofusin 1 (MFN1) and MFN2 are mitochondrial outer membrane GTPases responsible for mitochondrial outer membrane fusion [55]. Mitochondrial fission 1 protein (FIS1) is involved in mitochondrial fission via DRP1 binding, a fission protein activated by cellular stress and implicated in calcium uptake [56]. While MFN1 and FIS1 levels were comparable to controls, MFN2 and DRP1 levels were elevated, indicating a disrupted balance between mitochondrial fusion and fission (Fig. 1B, C). Despite no observed changes in lactate dehydrogenase (LDH) levels (Fig. 1C), the morphological changes in mitochondria hinted at underlying mitochondrial damage. Elevated levels of superoxide dismutase 1 (SOD1) in PC patients were consistent with increased reactive oxygen species (ROS) (Fig. 1B). To further investigate mitochondrial recycling, we assessed ATG4B levels, finding them to be higher in PC patients, supporting the hypothesis of enhanced mitophagy as a response to mitochondrial dysfunction (Fig. 1A). We also quantified the morphological changes occurring on the mitochondria of the PC patients which revealed severe morphological and mitochondrial number changes in the cells (Fig. 1D).

### Diminished circulating cell-free mtDNA content in PC patients

**Fig. 2:**
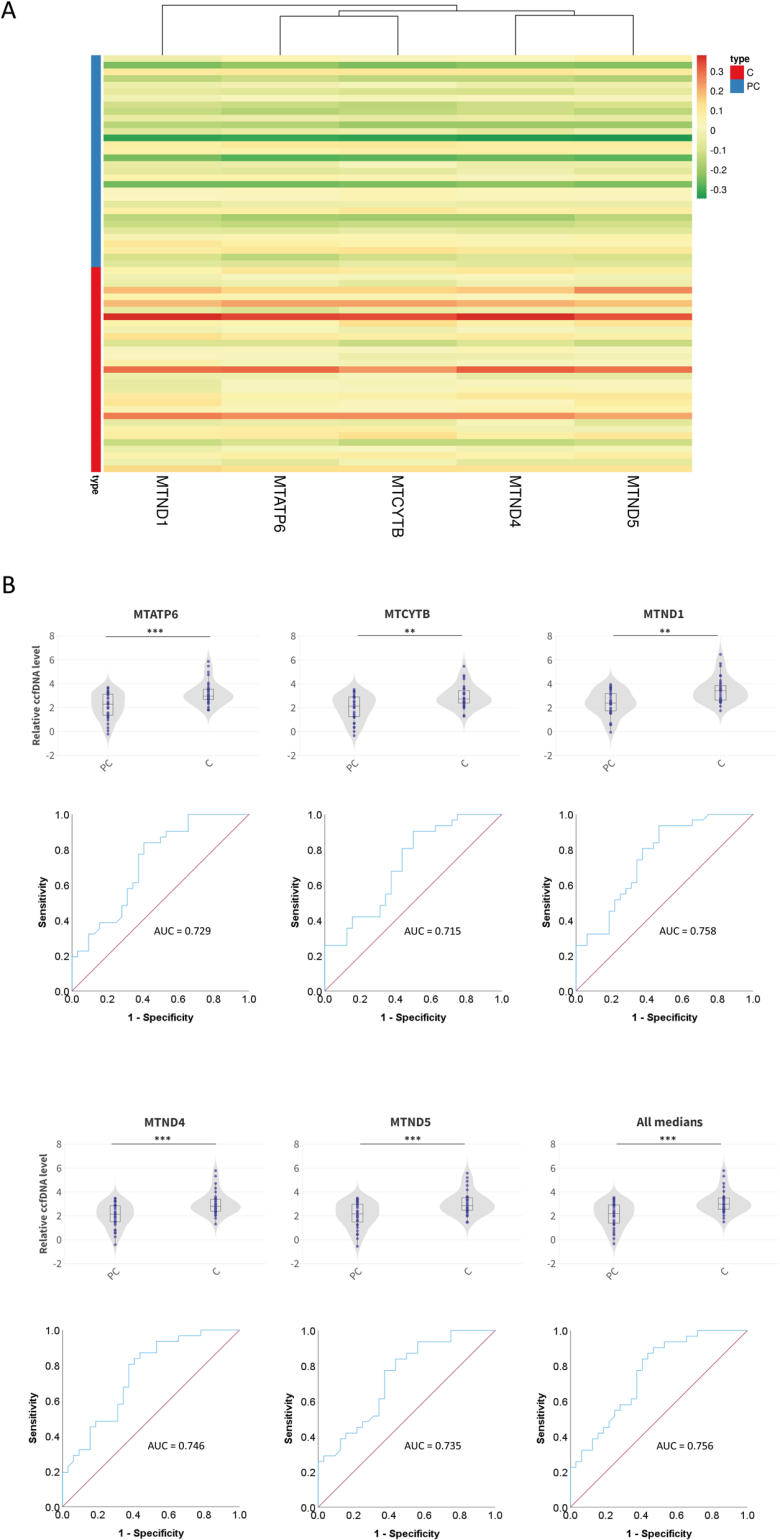
Quantitative analysis of ccf-mtDNA content in patients with post-COVID-19 (PC) syndrome and control participants. **A** Heatmap displaying the levels of ccf-mtDNA for five mitochondrial genes (*MTATP6*, *MTCYTB*, *MTND1*, *MTND4*, *MTND5*) in post-COVID-19 (PC, blue) and control (C, red) individuals. **B** Violin plots (first and third rows) showing the distribution of ccf-mtDNA levels for each mitochondrial gene, alongside receiver operating characteristic (ROC) curves (second and fourth rows) which evaluate the diagnostic potential of ccf-mtDNA measurements in distinguishing between the PC and C groups

We developed a standardized qPCR method to measure specific mitochondrial DNA (mtDNA) content in the plasma of PC and healthy volunteers. The study included 32 PC and 31 control participants. We quantified *MTATP6-*, *MTCYTB-*, *MTND1-*, *MTND4-*, and *MTND5-*specific plasma ccf-mtDNA content. The selection of these genes ensured comprehensive coverage of the mitochondrial genome, providing a robust evaluation of mitochondrial DNA integrity and quantity. Our findings revealed a significant reduction in ccf-mtDNA content in PC patients compared to healthy controls, indicating potential mitochondrial recycling dysfunction (Fig. 2A, B). To enhance the robustness of our results, we computed the median values from the individual ccf-mtDNA measurements and consolidated them into a single comprehensive dataset (denoted as “all medians”). This aggregate analysis reaffirmed a substantial reduction in mtDNA levels among PC patients relative to healthy controls. The significance of these observations was further substantiated by statistical analyses, which revealed a consistent pattern of diminished ccf-mtDNA levels across the PC cohort (Fig. 2A). The receiver operating characteristic (ROC) curves for each mitochondrial gene region confirmed the diagnostic utility of ccf-mtDNA, with area under the curve (AUC) values ranging from 0.715 to 0.758, suggesting moderate to high accuracy in distinguishing between the two cohorts (Fig. 2B).

## Discussion

**Fig. 3:**
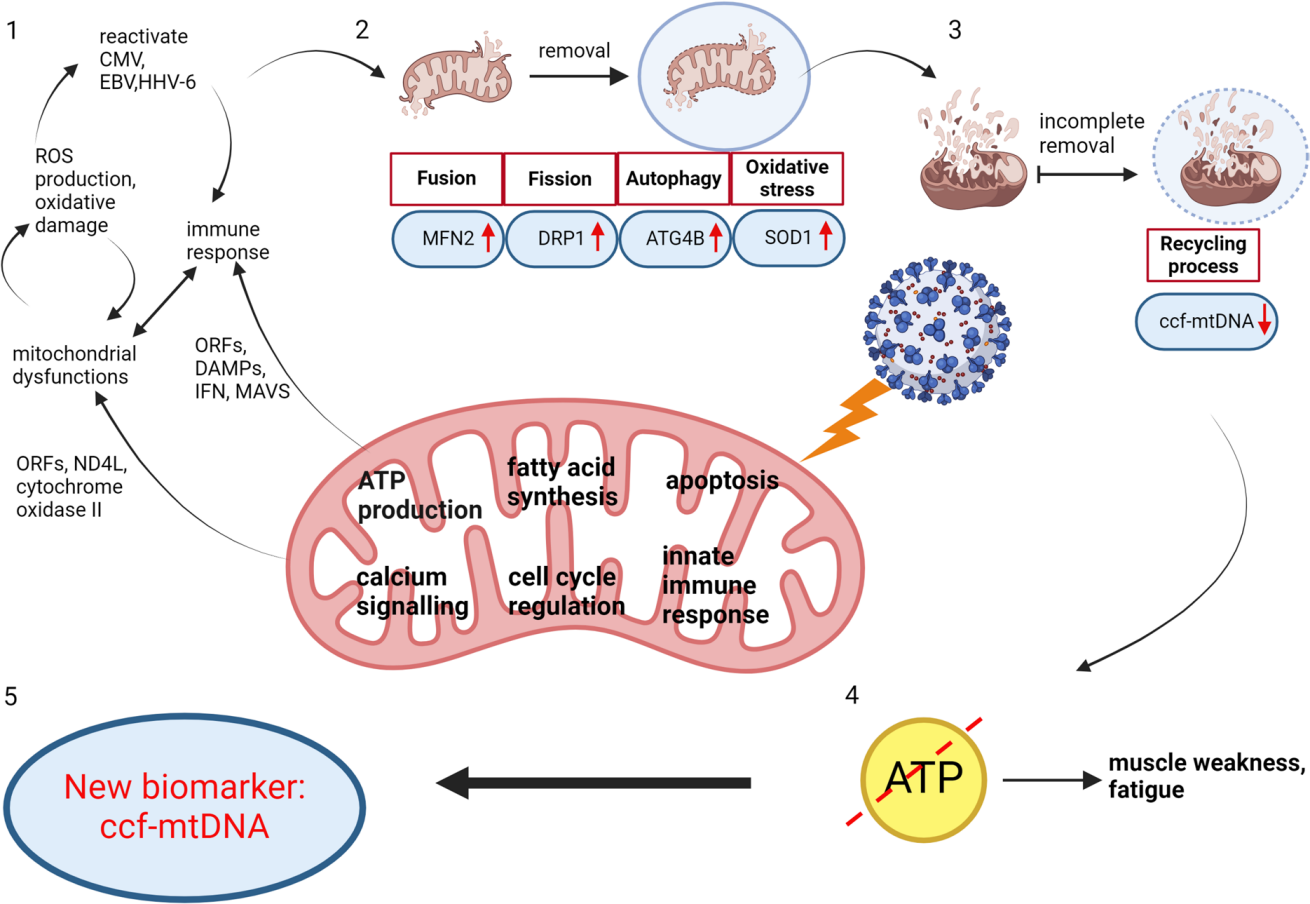
Mechanisms and consequences of mitochondrial damage and dysfunction in the pathogenesis of Long COVID. This schematic illustrates the cascade of events leading from initial SARS-CoV-2 infection to persistent mitochondrial dysfunction and its systemic effects. The diagram highlights key steps: (1) initial mitochondrial damage through direct viral interaction and immune-mediated responses; (2) activation of mitophagy in an attempt to clear damaged mitochondria; (3) persistent mitochondrial dysfunction due to incomplete removal of damaged mitochondria, evidenced by reduced ccf-mtDNA levels; (4) resultant systemic effects contributing to the symptomatology of Long COVID; (5) utilization of ccf-mtDNA as a diagnostic and monitoring tool to assess the extent of mitochondrial dysfunction. Each component integrates findings from the current study, emphasizing the role of mitochondrial damage in the pathogenesis of Long COVID

This study aimed to elucidate the role of mitochondrial dysfunction in Long COVID by examining mitochondrial structure, dynamics, and DNA content in PC patients compared to healthy controls. Our findings reveal significant mitochondrial abnormalities in PC patients, including compromised mitochondrial integrity, an imbalance in proteins that regulate mitochondrial fusion and fission, and reduced ccf-mtDNA content. Notably, the altered levels of assessed mitochondrial biomarkers in PC patients suggest mitochondrial malfunction and disrupted mitochondrial dynamics, potentially underpinning the persistence of post-COVID symptoms (Fig. 3).

Mitochondria are versatile cellular organelles that play a central role in numerous biochemical pathways, including ATP production and fatty acid synthesis, calcium signaling, cell cycle regulation, apoptosis, and innate immune response [57]. The observed mitochondrial structural changes in PC patients, such as dilated cristae and enlarged mitochondria, indicate severe mitochondrial distress. These alterations can impact mitochondrial efficiency, leading to insufficient ATP production and an increase in ROS. The link between such structural abnormalities and the elevated levels of SOD1 underscores a heightened oxidative stress response in PC patients, a condition that can exacerbate cellular damage and prolong recovery from viral infections. The imbalance in mitochondrial dynamics highlighted by increased levels of MFN2 and DRP1 could be indicative of the cell’s attempt to maintain mitochondrial function by enhancing fusion and fission processes. However, these compensatory mechanisms may not suffice to restore normal mitochondrial function and could instead lead to further dysregulation of cellular energy metabolism. This dysregulation is critical in understanding the widespread energy deficiency experienced by PC patients, manifesting as chronic fatigue and muscular weakness. Accordingly, research has revealed impairments in mitochondrial respiration, bioenergetics, and gene expression within peripheral blood mononuclear cells of Long COVID patients [58–62]. These deficits suggest that diminished mitochondrial energy production may contribute to prevalent symptoms like fatigue and muscle weakness. Additionally, magnetic resonance spectroscopy has detected mitochondrial dysfunction in the muscle tissue and brains of those affected, supporting clinical observations of exercise intolerance and post-exertional malaise [63–67]. Additional support for the role of mitochondria in Long COVID is provided by biomarker studies. These studies have identified specific markers that indicate mitochondrial dysfunction, further linking it to the condition’s persistent symptoms. Elevated levels of circulating biomarkers indicative of oxidative stress and mitochondrial damage, such as F2-isoprostanes and malondialdehyde, PARylation along with decreased levels of antioxidants such as coenzyme Q10, have been documented in Long COVID patients [46, 48, 68–73]. These biomarkers underscore the role of oxidative stress in exacerbating mitochondrial dysfunction associated with Long COVID. The significant reduction in circulating ccf-mtDNA levels among PC patients suggests an impaired mitochondrial recycling process. This finding is crucial as it points to a potential systemic impact of mitochondrial dysfunction, which could extend beyond the initially infected cells to affect various tissues and organ systems. The diagnostic potential of ccf-mtDNA underscores its utility in identifying patients with Long COVID, where mitochondrial damage and dysfunction are pivotal to the condition’s pathogenesis.

The mechanisms by which SARS-CoV-2 induces mitochondrial damage are likely multifaceted. Direct interactions between viral proteins and mitochondrial components disrupt the normal function and dynamics of mitochondria [74, 75] and cause structural damage [44, 76–79]. It has become evident that viruses employ various mechanisms to target host cell mitochondria to support viral particles’ growth and survival, further weakening the host’s cellular immune response and enhancing cell death. Viral infection often results in the release of damage-associated molecular patterns (DAMPs) that activate the antiviral immune response [80]. mtDNAs belong to mitochondrial DAMPs which are released by damaged cells [81] contributing to a heightened state of systemic inflammation [81]. Additionally, it has been reported that SARS-CoV-2 infection increases ROS production, causing oxidative damage to mtDNA and proteins, further exacerbating mitochondrial dysfunction [48]. Indirectly, the inflammatory response and immune dysregulation triggered by the infection can exacerbate mitochondrial damage. These mechanisms together suggest that SARS-CoV-2 not only targets mitochondrial health directly but also creates a systemic environment that perpetuates mitochondrial and cellular dysfunction.

Mitochondria undergo coordinated fusion and fission cycles, leading to transient morphological adaptations essential for various molecular processes such as cell cycle control, immune function, mitochondrial quality control, and apoptosis [82]. Our results suggest that mitochondrial dysfunction in PC patients is associated with disruptions in pathways that regulate mitochondrial fusion–fission and mitophagy. These disorders can exacerbate metabolic imbalance, contributing to post-COVID-19 symptoms [83]. Notably, the mitochondrial dysfunction observed in Long COVID shares similarities with other post-viral syndromes such as myalgic encephalomyelitis/chronic fatigue syndrome (ME/CFS) [60, 84–87]. Drawing parallels between these conditions may illuminate common mechanisms and shared therapeutic targets, providing a broader context for understanding post-viral conditions.

The development of autoimmunity following COVID-19 [88–96], wherein the immune system mistakenly targets mitochondrial proteins [97] and other cellular components, could further exacerbate mitochondrial dysfunction [98]. This autoimmune response may contribute to the chronic persistence of symptoms such as fatigue, muscle weakness, and neurological impairments by continually undermining mitochondrial function and preventing recovery.

Moreover, the stress of the infection and subsequent immune system alterations may reactivate latent herpesviruses such as cytomegalovirus (CMV), Epstein-Barr virus (EBV), and human herpesvirus 6 (HHV-6) [99–114], all known to influence mitochondrial function. The reactivation of these viruses during or after COVID-19 can exacerbate mitochondrial damage, thereby contributing to the severity and persistence of Long COVID symptoms [99, 115], further complicating the clinical picture and potentially hindering recovery.

Mitochondrial dysfunction impacts various organs differently, which helps explain the wide range of symptoms associated with Long COVID. In the brain, it may contribute to neurological symptoms like “brain fog” and fatigue. In the heart, it can lead to energy deficits that manifest as cardiac symptoms such as arrhythmias. Additionally, the importance of mitochondria in vascular endothelial function cannot be overlooked [116–120], especially considering that SARS-CoV-2 exhibits endothelial trophism [17]. There is a growing body of literature suggesting that endothelial dysfunction plays a central role in the pathogenesis of both acute COVID-19 and Long COVID. The endothelium relies heavily on mitochondrial integrity for the regulation of vascular tone and maintenance of the blood–brain barrier [116–120]. Mitochondrial dysfunction in endothelial cells can lead to impaired production of nitric oxide, a critical vasodilator, thereby contributing to vascular stiffness, hypertension, and impaired blood flow to the brain, muscles, and heart. Moreover, endothelial mitochondrial damage might enhance the permeability of the blood–brain barrier, facilitating the influx of inflammatory mediators into the central nervous system. The resulting heightened inflammatory state in the brain can exacerbate neurological symptoms and may also contribute to the multisystem involvement seen in Long COVID. Thus, in Long COVID, mitochondrial dysfunction in the vasculature likely contributes to a range of manifestations, from vasodilator dysfunction to blood–brain barrier disruption. Additionally, immune responses triggered by factors released from damaged mitochondria may contribute to persisting inflammation and thereby to the development of post-COVID-19 conditions [121–123]. These effects collectively compound the complex symptomatology of Long COVID, linking systemic mitochondrial impairment with organ-specific clinical outcomes. The systemic nature of mitochondrial dysfunction thus serves as a unifying pathophysiological mechanism underlying the diverse and persistent symptoms observed in patients with Long COVID.

The insights gained from this study pave the way for exploring mitochondrial-targeted therapies as potential treatments for Long COVID [36]. Interventions that enhance mitochondrial function, including the use of mitochondrial-targeted antioxidants, lifestyle modifications like improved diet and exercise, and potentially pharmaceutical interventions, are under investigation [36]. These strategies aim to restore mitochondrial health [48, 49], which could alleviate the broad spectrum of Long COVID symptoms. Among them, several compounds with known mitochondrial protective effects, such as Q1067, MitoQ (NCT05373043), alpha-lipoic acid, nicotinamide riboside (NCT05703074), and resveratrol (NCT05601180), are currently under investigation in clinical trials [124–126]. Further research is needed to explore these therapeutic avenues and to validate the effectiveness of novel biomarkers for monitoring disease progression and treatment response.

In particular, identifying reliable biomarkers of mitochondrial dysfunction is critical [36]. In our study, we investigated the utility of plasma mtDNA content as a diagnostic tool for post-COVID-19 conditions. In contrast to our initial hypothesis that increased mitophagy would elevate ccf-mtDNA levels in patients with chronic symptoms, we observed lower ccf-mtDNA levels. This suggests that while mitochondrial clearance mechanisms are activated, they fail to completely remove damaged mitochondria. Supporting this, we noted differences in mitochondrial morphology and size between PC patients and controls, indicating persistent mitochondrial abnormalities despite active mitophagy. Importantly, the correlation between reduced ccf-mtDNA levels and symptom severity underscores its potential as a valuable biomarker for diagnosing and monitoring post-COVID-19 conditions, offering a promising means to differentiate between affected individuals and healthy controls and assess the extent of mitochondrial dysfunction. The development and validation of these and similar biomarkers could significantly improve the diagnosis and monitoring of Long COVID, aiding in the assessment of treatment efficacy and understanding disease progression [36].

In conclusion, our study has substantiated the pivotal role of mitochondrial dysfunction in the chronic manifestations of Long COVID [36]. As we further extended our understanding of these underlying mechanisms, it becomes clear that aging may play a significant modulatory role in these processes [17]. Aging is known to induce mitochondrial dysfunction across various cell types, contributing to the functional decline of these organs and rendering cells and mitochondria less resilient. This vulnerability may exacerbate the severity of mitochondrial damage observed in Long COVID, making the elderly particularly susceptible to prolonged and severe post-viral symptoms [17]. Therefore, it is imperative that future studies explore how aging influences mitochondrial dynamics in the context of Long COVID. Such research could provide insights into age-specific therapeutic interventions and preventive measures, ultimately aiding in the development of targeted strategies that not only improve the quality of life for older adults but also reduce the broader, long-term health impacts of the COVID-19 pandemic. By integrating insights from various medical disciplines and drawing parallels with other post-viral syndromes, we can enhance our management of Long COVID, paving the way for interventions that address the multifaceted aspects of this condition in an age-sensitive manner.

## Data Availability

The data described in the manuscript may be made available upon request pending application and approval by the corresponding author.
